# Diagnostic Performance of Antigen Rapid Diagnostic Tests, Chest Computed Tomography, and Lung Point-of-Care-Ultrasonography for SARS-CoV-2 Compared with RT-PCR Testing: A Systematic Review and Network Meta-Analysis

**DOI:** 10.3390/diagnostics12061302

**Published:** 2022-05-24

**Authors:** Sung Ryul Shim, Seong-Jang Kim, Myunghee Hong, Jonghoo Lee, Min-Gyu Kang, Hyun Wook Han

**Affiliations:** 1 Department of Health and Medical Informatics, Kyungnam University College of Health Sciences, Changwon 51767, Korea; ryul01@korea.ac.kr; 2 Department of Nuclear Medicine, Pusan National University Yangsan Hospital, Yangsan 50615, Korea; growthkim@pusan.ac.kr; 3 Department of Nuclear Medicine, College of Medicine, Pusan National University, Yangsan 50615, Korea; 4 BioMedical Research Institute for Convergence of Biomedical Science and Technology, Pusan National University Yangsan Hospital, Yangsan 50615, Korea; 5 Department of Biomedical Informatics, CHA University School of Medicine, CHA University, Seongnam 13488, Korea; mhhong99486@gmail.com; 6 Institute for Biomedical Informatics, School of Medicine, CHA University, Seongnam 13488, Korea; 7 Department of Internal Medicine, Jeju National University Hospital, Jeju National University School of Medicine, Jeju 63241, Korea; lovlet@paran.com; 8 Department of Internal Medicine, Chungbuk National University College of Medicine, Chungbuk National University Hospital, Cheongju 28644, Korea; irreversibly@gmail.com; 9 Institute of Basic Medical Sciences, School of Medicine, CHA University, Seongnam 13488, Korea

**Keywords:** SARS-CoV-2, COVID-19, rapid antigen diagnostic test, RT-PCR, imaging diagnostic test, computed tomography, ultrasonography, meta-analysis, systematic review

## Abstract

(1) Background: The comparative performance of various diagnostic methods for severe acute respiratory syndrome coronavirus-2 (SARS-CoV-2) infection remains unclear. This study aimed to investigate the comparison of the 3 index test performances of rapid antigen diagnostic tests (RDTs), chest computed tomography (CT), and lung point-of-care-ultrasonography (US) with reverse transcription-polymerase chain reaction (RT-PCR), the reference standard, to provide more evidence-based data on the appropriate use of these index tests. (2) Methods: We retrieved data from electronic literature searches of PubMed, Cochrane Library, and EMBASE from 1 January 2020, to 1 April 2021. Diagnostic performance was examined using bivariate random-effects diagnostic test accuracy (DTA) and Bayesian network meta-analysis (NMA) models. (3) Results: Of the 3992 studies identified in our search, 118 including 69,445 participants met our selection criteria. Among these, 69 RDT, 38 CT, and 15 US studies in the pairwise meta-analysis were included for DTA with NMA. CT and US had high sensitivity of 0.852 (95% credible interval (CrI), 0.791–0.914) and 0.879 (95% CrI, 0.784–0.973), respectively. RDT had high specificity, 0.978 (95% CrI, 0.960–0.996). In accuracy assessment, RDT and CT had a relatively higher than US. However, there was no significant difference in accuracy between the 3 index tests. (4) Conclusions: This meta-analysis suggests that, compared with the reference standard RT-PCR, the 3 index tests (RDTs, chest CT, and lung US) had similar and complementary performances for diagnosis of SARS-CoV-2 infection. To manage and control COVID-19 effectively, future large-scale prospective studies could be used to obtain an optimal timely diagnostic process that identifies the condition of the patient accurately.

## 1. Introduction

The emergence of severe acute respiratory syndrome coronavirus-2 (SARS-CoV-2) in late 2019 caused the coronavirus disease 2019 (COVID-19) global pandemic, with more than 293 million infections worldwide [1]. In order to reduce the spread of the virus and treat COVID-19 patients in a timely manner, accurate and rapid detection of SARS-CoV-2 infection is required [2].

The reverse transcription-polymerase chain reaction (RT-PCR) assay is regarded as the gold standard laboratory technique for identifying SARS-CoV-2 [3]. RT-PCR has a high diagnostic accuracy for SARS-CoV-2, with a sensitivity ranging from 71 to 98% and a specificity of 100% [4,5]. Although RT-PCR has high diagnostic performance, it has some potential disadvantages such as the need for trained laboratory expertise, expensive devices, long learning periods, and variabilities of diagnostic accuracy over the disease course [6,7,8,9].

The increased global burden of SARS-CoV-2 infection necessitates the development of rapid, accurate, antigen tests for detection of SARS-CoV-2 viral proteins in respiratory samples. These rapid antigen tests (RDTs) are important diagnostic tools in preventing the spread of SARS-CoV-2 infection [10,11]. A recent meta-analysis comparing the diagnostic test accuracy of SARS-CoV-2 infection showed that the overall sensitivity of RDTs was lower than that of the RT-PCR assay [12]. However, RDTs showed high sensitivity with a Ct-value ≤ 25 and were applicable within 5 days of symptom onset for subjects in the community [12]. Because COVID-19 is an infectious disease that causes inflammation in the respiratory system, chest imaging could be used both for diagnosis and to define the extent of diseases and enable accurate identification of changes during the disease process. In a recent Chinese study involving over 1000 patients, computed tomography (CT) was found to have high sensitivity for detecting COVID-19 infection, suggesting that CT could be a useful COVID-19 infection screening tool in epidemic areas [13]. In addition, when RT-PCR is not available, chest CT has been proposed as an alternative diagnostic method for COVID-19 [14]. Another imaging modality for COVID-19, lung point-of-care-ultrasonography (US) has been proposed as a screening tool; several studies have suggested that US has potential in the diagnosis of COVID-19 based on weak preliminary evidence [15,16,17].

Despite publication of abundant studies, there is no comprehensive research simultaneously comparing the diagnostic performance of the optimal diagnostic methods of COVID-19 at the same time. In this respect, network meta-analysis (NMA) of diagnostic test accuracy (DTA) associated is a new approach that provides an overall assessment of test accuracy [18]. Based on current evidence, this new approach provides useful information to stakeholders and policymakers who promote decision-making processes to enhance the value of testing in various diagnostic environments and implement it in clinical practice [19].

Therefore, we conducted an NMA using published direct comparison studies with 2 or more index tests, aiming to investigate and compare the performance of 3 index tests, RDT, chest CT, and lung US, for COVID-19 infection. The study aims to provide additional evidence-based evidence on guidelines for appropriate use of these index tests.

## 2. Materials and Methods

The protocol of this systematic review and NMA was registered at PROSPERO (CRD42021286536). Reporting of this NMA is based on the preferred reporting items for systematic reviews and meta-analyses extension for NMA of healthcare interventions guidelines [20].

### 2.1. Data Sources and Literature Searches

We systematically retrieved data from electronic literature searches of PubMed and Cochrane Library using MeSH headings and text words from 1 January 2020, to 1 April 2021. The subject headings included those related to the population (patients with suspected SARS-CoV-2 infection), index tests (RDTs, chest CT, and lung US), and reference standard (RT-PCR). The search terms were organized using Boolean operators (AND, OR, NOT). The searches were limited to human studies and had no restriction on language or study type. The same search manner was applied to EMBASE using Emtree (subject headings). Additional studies were screened by two independent investigators (SR Shim and HW Han) through manual search of clinical trial databases and previous study references (Table S2).

### 2.2. Study Selection

The study inclusion criteria were (1) patients suspected of having SARS-CoV-2 infection; (2) index tests performed for diagnosis of SARS-CoV-2 infection; and (3) the reference standard was RT-PCR; (4) the outcomes were sensitivity, specificity, positive predictive value (PPV), negative predictive value (NPV), accuracy, and area under the curve (AUC). Publications without original data such as review articles, case reports, conference abstracts, editorials, letters, and guidelines were excluded. Using predefined inclusion criteria, two investigators (SR Shim and SJ Kim) independently previewed the titles and abstracts of all the articles. All the investigators independently examined the full-text articles to determine whether they met the inclusion criteria. Furthermore, the same authors (SR Shim and HW Han) extracted data using a data extraction form. The final inclusion of each paper was determined through evaluation and discussion by all investigators. The references and data included were cross-checked to maintain the integrity of NMA and the absence of overlapping data.

### 2.3. Data Extraction and Quality Assessment

Trial characteristics of interest were (1) study information (first author, year of publication, country), (2) study design (prospective or retrospective), (3) patient characteristics (number of patients, mean age, sex), and (4) technical aspects. Each study was calculated to retrieve the sensitivity, specificity, PPV, NPV, and accuracy of index tests according to the reference standard. Studies containing incomplete information were excluded, and only studies that provided complete outcomes were included in the final meta-analysis. The overall quality assessment was critically appraised by discussion of all authors based on quality assessment of diagnostic accuracy studies (QUADAS-2) [21]. Through the discussion of all authors, discrepancies between individual researchers were resolved. QUADAS-2 consists of four domains: patient selection, index test, reference standard, and flow and timing. Each domain is evaluated in terms of risk of bias, and the three domains of patient selection, index test, and reference standard are assessed in terms of applicability. STATA 14.0 (StataCorp, College Station, TX, USA) was used to assess the quality.

### 2.4. Data Analysis

To identify the performance of various methods for diagnosis of SARS-CoV-2 infection compared with RT-PCR, we used an approach incorporating two meta-analysis methodologies. First, pairwise meta-analysis using DTA was conducted for direct comparison of various diagnostic methods by RT-PCR. The bivariate random-effects model was used for DTA analysis with pooling of the diagnostic performance measures across studies, as well as comparison between index tests. By incorporating any possible correlation between sensitivity and specificity, the bivariate model estimates logit-transform sensitivity and specificity pairs in the studies [18]. The diagnostic accuracy of each index test was assessed using a hierarchical summary receiver operating curve (HSROC) and AUC. The I2 statistic and Cochrane’s Q test were used to assess heterogeneity among the outcomes of the included studies in this meta-analysis. R version 4.1.1 was used for all statistical analyses (R Foundation for Statistical Computing, Vienna, Austria). Statistical significance was defined as a two-sided α less than 0.05.

To compare the three index tests (RDT, CT, and US) with RT-PCR, we used Bayesian NMA with the “gemtc” package in R software according to a Bayesian method [22,23,24]. First, a prior distribution was selected. Second, the likelihood was calculated from the present data, and a Bayesian hierarchical model was created in NMA. Third, the prior distribution and likelihood were fed into a Markov chain Monte Carlo (MCMC) simulation, and the distribution with the best convergence of the posterior distribution was chosen. The MCMC simulation was used to determine the probability of a stable distribution and the area under the posterior distribution function. Finally, the posterior distribution was used to perform statistical reasoning for the treatment effect.

We performed node-splitting assessments to determine the association between the direct and indirect evidence for the consistency test. The surface under the cumulative ranking curve (SUCRA) was used to calculate the probability of each index test being the most effective diagnostic method based on a Bayesian approach using probability values to facilitate interpretation of diagnostic performance; the larger the SUCRA value was, the higher the rank of the intervention [25].

In addition, sensitivity analysis was used to determine how different RDT sampling methods (nasopharyngeal swab and nasal, saliva, or throat swab) affected NMA and SROC.

Publication bias was assessed with funnel plots using standard errors and mean differences of treatment effect. In the funnel plot, asymmetry indicates publication bias, but the shape of the plot depends on the choice of trial arms.

## 3. Results

### 3.1. Study Selection

**Figure 1:**
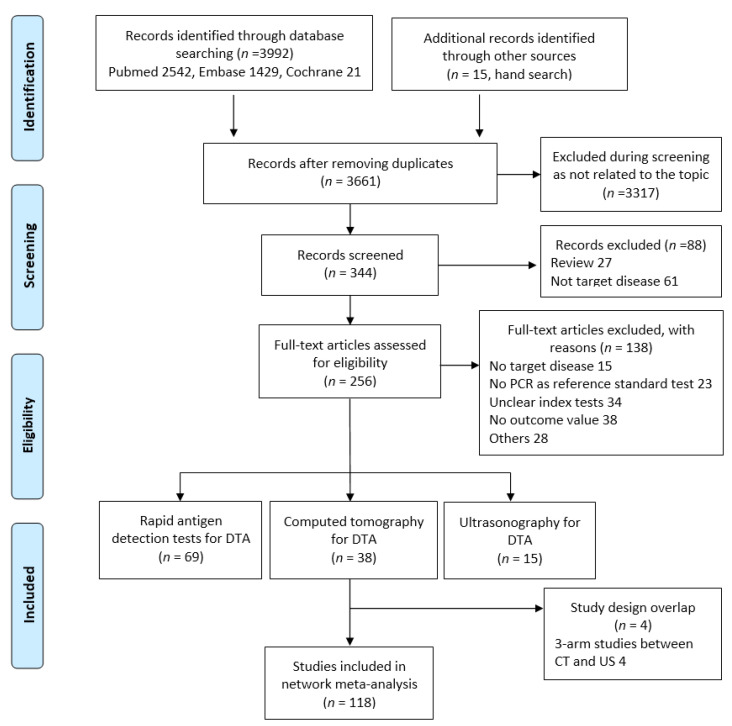
PRISMA flow diagram of study selection process.

Systematic search identified a total of 3992 articles from electronic databases (PubMed, 2542; Cochrane, 21; and EMBASE, 1429). After exclusion of 346 studies containing overlapping data or appearing in more than one database and after screening the titles and abstracts, 3317 studies that did not meet the inclusion criteria were excluded. After intensive screening, an additional 88 papers that did not contain original data or target diseases were eliminated, leaving 256 studies eligible for intensive screening. Of the remaining studies, 138 were further excluded for the following reasons: no target disease (*n* = 15), no RT-PCR as the reference standard (*n* = 23), unclear index tests (*n* = 34), no outcome value (*n* = 38), and others (*n* = 28). Finally, 118 studies including 69445 participants met our selection criteria for NMA, among which 69 RDT, 38 CT, and 15 US were included in the pairwise meta-analysis for DTA. There was overlap in the design of four 3-arm studies between CT and US (Figure 1).

The characteristics and detailed information of the 118 studies included are described in Table S1. All studies have the common comparator of RT-PCR as the reference standard. Most of the studies have been implemented in North America or Europe. Nasopharyngeal swab of RDT sampling was used for diagnosis of SARS-CoV-2 in 54 studies, and nasal, saliva, or throat swab was used in 15. Most of the populations comprised only adults or adults and children, and there were only two groups including children (Table S1).

### 3.2. Quality Assessment

All authors critically appraised the 118 studies selected using the critical criteria of the QUADAS-2 assessment (Figure S1). For Applicability concerns, the included studies were matched with the review questions, especially Reference Standard and Patients’ Selection were 100% (118/118) of low and 89% (105/118) of low or unclear applicability concerns. For risk of bias, however, 78% (92/118) did not have a clear description (unclear or high risk of bias) of the patient selection method, and only 14% (10/69) of RDT was reported properly.

### 3.3. Pairwise Meta-Analysis for DTA

**Figure 2:**
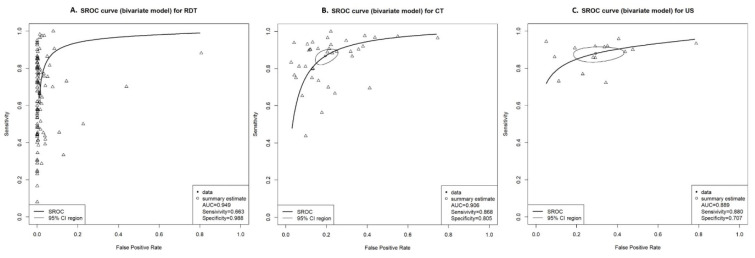
Summary receiver operating characteristic (SROC) curve of three index tests (RDTs, chest CT, and lung US) compared to the reference standard RT-PCR in the diagnosis of SARS-CoV-2 infection. The 95% CI region of RDT is not clearly formed due to its high heterogeneity.

A direct pairwise comparison of the 3 diagnostic methods for detection of SARS-CoV-2 infection was performed. The overall estimated sensitivity, specificity, and AUC with 95% confidence interval (CI) of DTA were showed (Figure 2). CT and US had a high sensitivity of 0.868 (95% CI, 0.831–0.898) and 0.880 (95% CI, 0.843–0.909), respectively. RDT had a high specificity of 0.988 (95% CI, 0.984–0.992) and AUC of 0.949 (95% CI, 0.922–0.960). In summary ROC (SROC), because CT and US had symmetrical sensitivity and specificity, a narrow 95% confidence area was formed, and these two test methods showed low heterogeneity. On the other hand, RDT has asymmetrical low sensitivity and high specificity, preventing formation of a confidence contour and indicating high heterogeneity (Figure 2).

### 3.4. Network Meta-Analysis

**Figure 3:**
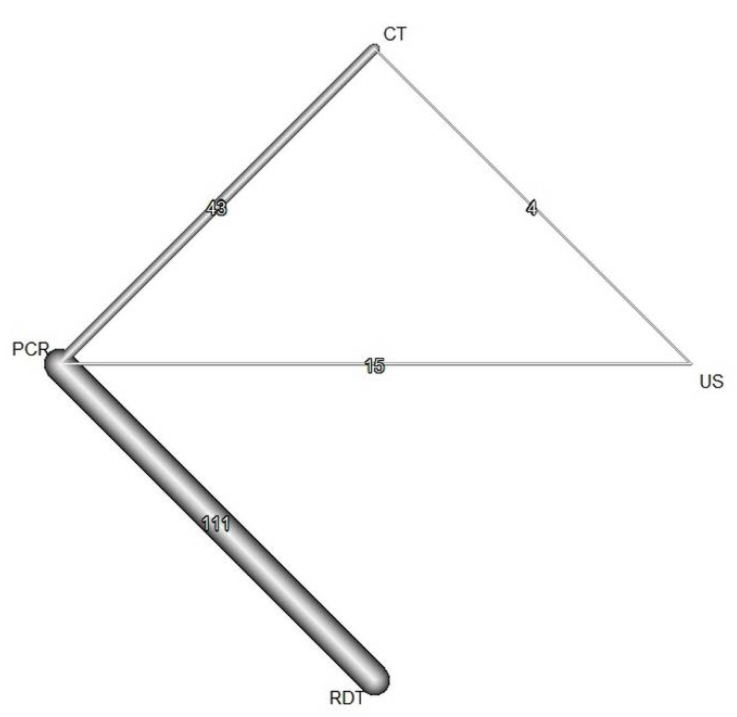
Network plots for network meta-analysis. The width of lines is proportional to the number of trials comparing each pair of treatments. RT-PCR = reverse transcription-polymerase chain reaction. RDT = rapid antigen diagnostic tests. CT = chest computed tomography. US = lung point-of-care-ultrasonography.

The network plot of diagnostic tests showed 4 nodes (3 index tests plus RT-PCR as The network plot of diagnostic tests showed 4 nodes (3 index tests plus RT-PCR as the reference standard) and 174 comparisons (Figure 3). CT and US had a high sensitivity of 0.852 (95% credible interval (CrI), 0.791–0.914) and 0.879 (95% CrI, 0.784–0.973), respectively. RDT showed high specificity of 0.978 (95% CrI, 0.960–0.996).

**Figure 4:**
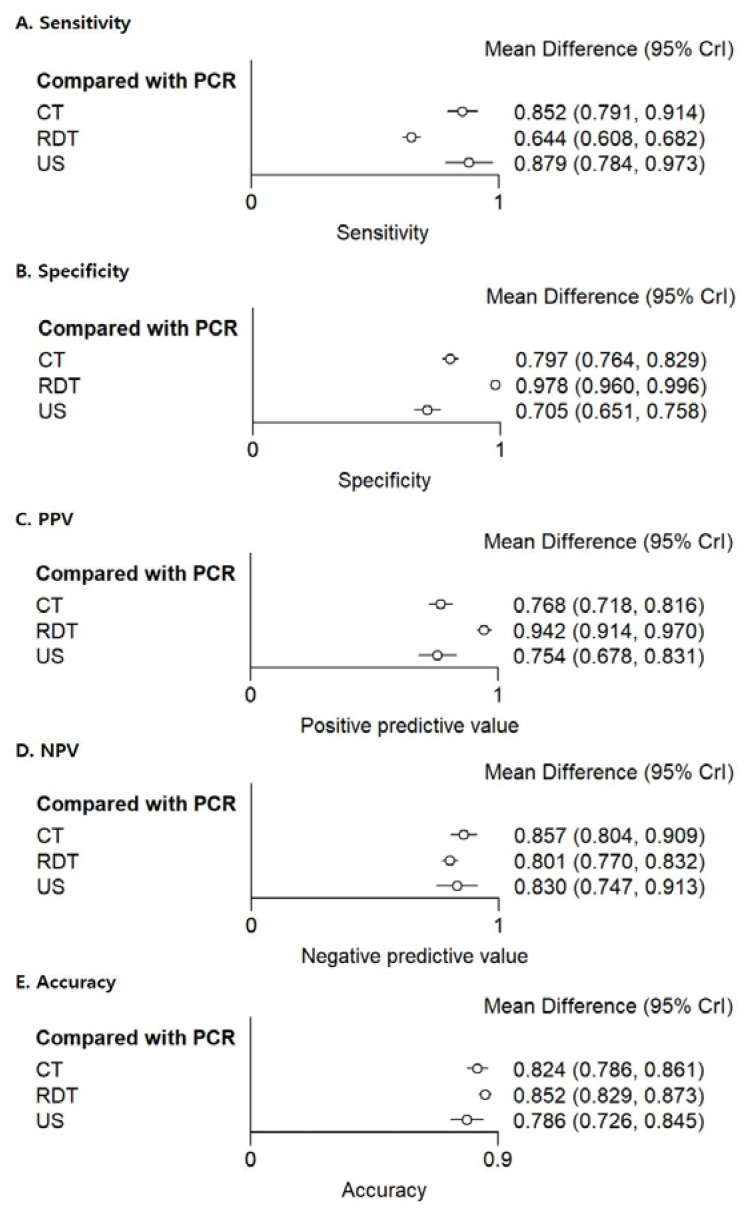
Forrest plots for three index tests (RDTs, chest CT, and lung US) compared to the reference standard RT-PCR in the diagnosis of SARS-CoV-2 infection. PPV = positive predictive value. NPV = negative predictive value.

In accuracy, RDT and CT have a relatively higher value of 0.852 (95% CrI, 0.829–0.873) and 0.824 (95% CrI, 0.786–0.861), respectively, than US with a value of 0.786 (95% CrI, 0.726–0.845) (Figure 4). There was no significant difference in accuracy between the 3 index tests (*p* > 0.05). The index tests for NMA were analyzed for consistency using the node-splitting method, and all *p*-values of the assumption of consistency between direct and indirect evidence of all outcomes were satisfied (all *p* > 0.05). Therefore, the current NMA can be calculated interchangeably with direct and indirect treatment comparisons.

Table S3 shows the SUCRA values of the diagnostic performance of the 3 methods for detection of SARS-CoV-2 infection, which indicated that US and CT ranked first in terms of sensitivity and NPV, and RDT ranked first in specificity and PPV.

The results of comparative performance of RDT sampling methods between the nasopharyngeal swab and the nasal, saliva, or throat swab are shown in Figure S2. The sensitivity of RDT decreased to 0.577 (95% CrI, 0.510–0.643) in the nasal, saliva, or throat swab compared with 0.659 (95% CrI, 0.620–0.697) in the nasopharyngeal swab. In the two SROCs, individual studies were distributed according to a wide range of sensitivity, indicating high heterogeneity (Figure S2).

### 3.5. Publication Bias

**Figure 5:**
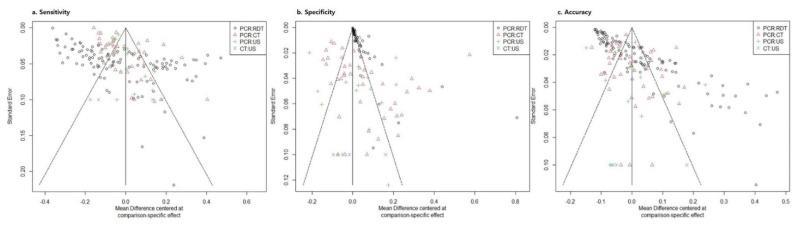
Funnel plots for diagnostic comparisons. RT-PCR = reverse transcription-polymerase chain reaction. RDT = rapid antigen diagnostic tests. CT = chest computed tomography. US = lung point-of-care-ultrasonography.

Figure 5 shows the results of publication bias or small-study effect in the 118 studies included in NMA. In all summary statistics, individual studies of CT and US were symmetrically distributed with respect to combined effect size and standard error of the graph. However, the distribution of RDT studies was scattered asymmetrically, especially sensitivity, NPV, and accuracy. Thus, RDT was estimated to have a publication bias in this NMA (Figure 5).

## 4. Discussion

The results of this NMA showed no significant difference in accuracy between RDTs, chest CT, and lung US with reference to RT-PCR. The SUCRA values of the diagnostic performance of the 3 methods for detection of SARS-CoV-2 infection indicated that US ranked first in terms of sensitivity, CT ranked first in NPV, and RDT ranked first in specificity, PPV, and accuracy. Considering the most up-to-date scientific knowledge, this is the first NMA and systematic review to compare diagnostic methods of viral antigen tests, viral RNA tests, and imaging modalities for SARS-CoV-2 infection. In addition, the comparative diagnostic performance of RDT sampling methods between the nasopharyngeal swab and the non-nasopharyngeal swab showed lower sensitivity of 0.577 of RDT in the non-nasopharyngeal swab compared with 0.659 in the nasopharyngeal swab.

RT-PCR is generally considered a gold standard diagnostic method for COVID-19 infection. However, due to the time-intensive procedures, it has potential limits, and the accuracy may vary depending on the techniques used by different laboratories. Nonetheless, the availability of RT-PCR kits may be limited, especially in developing countries, and positivity for SARS-CoV-2 infection is dependent on a number of factors including quality of RT-PCR kits, sampling sites and volume, transportation, and storage, as well as laboratory test conditions and personal operation techniques [26,27]. To overcome the disadvantages of RT-PCR, RDTs have been developed to detect viral proteins of SARS-CoV-2 in respiratory samples [28]. However, studies have reported conflicting results of RDTs for diagnosing SARS-CoV-2 infection [12,29,30,31]. Some studies have shown that RDTs have high sensitivity and specificity for diagnosis of SARS-CoV-2 infection [29,30,31]. However, other studies have reported that RDTs have imperfect diagnostic accuracy for SARS-CoV-2 infection, and diagnostic performance might be inferior to that of RT-PCR [12]. Despite these controversial diagnostic performances, the use of RDTs is recommended in symptomatic patients, and sensitivities might be highest in the first week of illness when viral loads are higher [12,29,30,31].

During the COVID-19 pandemic, chest CT was suggested as a diagnostic method for SARS-CoV-2 infection. The guideline of Diagnosis and Treatment of Pneumonitis Caused by 2019-nCoV (sixth version) recommended chest CT as an effective tool to screen patients suspected of having the disease [32]. According to a recent study comparing the diagnostic performance of chest CT with SARS-CoV-2 RT-PCR using the COVID-19 Reporting and Data System classification system (CO-RADS), chest CT with CO-RADS could be used for triage due to its good diagnostic performance in symptomatic individuals [33]. In addition, incidental detection of CO-RADS 3 or greater in asymptomatic people should prompt testing for respiratory pathogens [33]. The usefulness of chest CT for detecting COVID-19 has also been reported in other meta-analyses [34,35]. Mair et al. found that chest CT had a higher sensitivity than RT-PCR for detecting COVID-19 infection. However, the specificity of chest CT is relatively low because CT results might overlap with those of other viral infections. Thus, they concluded that chest CT is less likely to replace RT-PCR as the gold standard test [34].

Other studies showed that chest CT provides greater sensitivity in detecting COVID-19 infection, especially in areas with a severe epidemic status [35]. They assumed that, given emergency disease control, chest CT could provide a fast, convenient, and effective way to recognize patients with suspected infection early to contribute to management of the epidemic [35].

However, chest CT has major drawbacks of ionizing radiation and limited availability of equipment because of the high cost. Thus, lung US has been proposed as a screening tool for COVID-19 infection, and may offer some benefits due to its cost-effectiveness, portability, and ability to provide real-time data [36,37]. The disease activity of lung US in patients with SARS-CoV-2 infection correlated well with chest CT findings [38]. Brenner et al. suggested that because medical resources are limited during the explosive increase in infectious disease patients, lung US screening for SARS-CoV-2 can well identify which patients should undergo RT-PCR tests or whether additional tests are unnecessary [39]. According to a recent meta-analysis of chest imaging for COVID-19 diagnosis, chest CT has high sensitivity and moderate specificity, while lung US has high sensitivity but low specificity [40]. Thus, they suggested that chest CT and lung US are more useful in excluding COVID-19 than in distinguishing SARS-CoV-2 infection from other causes of respiratory diseases [40]. In the era of pandemic SARS-CoV-2 infection, thoracic imaging modalities such as chest CT and lung US could be useful strategies for identifying those who are COVID-19 infected. This would allow for better patient management such as isolation precautions; strategies for contact tracing and quarantine; admission to hospital, specialized facility, and intensive care unit; or initiation of standard guidelines and implementation of public health strategies to prevent SARS-CoV-2 infection from spreading. In addition, these modalities could be used to identify COVID-19 patients who need emergency operation without delay in waiting for the results of RT-PCR for SARS-CoV-2 infection.

Another major issue with the diagnostic performance of RT-PCR and RDTs is the sampling sites of respiratory samples of SARS-CoV-2 infection in patients. As shown in the current study, the non-nasopharyngeal swab showed inferior diagnostic accuracy compared with nasopharyngeal swab. Contrary to the current study, a recent meta-analysis suggested that saliva nucleic acid amplification testing (NAAT) is an attractive alternative to nasopharyngeal swab NAAT and can significantly bolster massive testing efforts [41]. Another study found that pooled nasal and throat swabs have higher diagnostic performance for SARS-CoV-2 infection in ambulatory care than the gold-standard nasopharyngeal swabs. However, use of throat swabs is not recommended because they show much lower sensitivity and PPV [42].

The present NMA had some limitations. Most studies included in the current investigation compared the diagnostic accuracy of RT-PCR and RDTs for SARS-CoV-2 infection. Fifteen studies compared chest RT-PCR and US and only 4 studies compared chest CT and US for detection of SARS-CoV-2 infection. Second, there were significant variabilities in the chest CT and lung US protocols used as well as the interpretation criteria of thoracic imaging modalities for the definition of a positive scan in the studies included. Despite these limitations, we believe the results of this NMA provide a useful reference framework for overall interpretation of SARS-CoV-2 testing results using diagnostic methods of viral antigen tests, viral RNA tests, and imaging modalities.

## 5. Conclusions

This present meta-analysis showed that, with reference to RT-PCR as the gold standard, 3 index tests (RDTs, chest CT, and lung US) showed similar and complementary diagnostic performances for diagnosis of SARS-CoV-2 infection. To manage and control COVID-19 effectively, future large-scale prospective studies are required to design an optimal timely diagnostic process for identifying the condition in patients accurately.

## Data Availability

Data is contained within the article or Supplementary Materials.

## Supplementary Materials

The following supporting information can be downloaded at: https://www.mdpi.com/article/10.3390/diagnostics12061302/s1, Figure S1. Quality Assessment of Diagnostic Accuracy Studies 2 (QUADAS-2) Study Quality Summary; Figure S2. Network meta-analysis forest plot and SROC between nasopharyngeal swab and other swab for RDT; Table S1. Characteristics of the included studies; Table S2. Search queries; Table S3. SUCRA Values of 3 Different methods for diagnosis of SARS-CoV-2 infection by PCR. List of studies included in the analysis, see [19,28,39,43,44,45,46,47,48,49,50,51,52,53,54,55,56,57,58,59,60,61,62,63,64,65,66,67,68,69,70,71,72,73,74,75,76,77,78,79,80,81,82,83,84,85,86,87,88,89,90,91,92,93,94,95,96,97,98,99,100,101,102,103,104,105,106,107,108,109,110,111,112,113,114,115,116,117,118,119,120,121,122,123,124,125,126,127,128,129,130,131,132,133,134,135,136,137,138,139,140,141,142,143,144,145,146,147,148,149,150,151,152,153,154,155,156,157].
