# Supplementary material for: Diagnostic Performance of Antigen Rapid Diagnostic Tests, Chest Computed Tomography, and Lung Point-of-Care-Ultrasonography for SARS-CoV-2 Compared with RT-PCR Testing: A Systematic Review and Network Meta-Analysis
Source: Diagnostics (Basel). 2022 May 24;12(6):1302. doi: 10.3390/diagnostics12061302 (PMC9222155; doi:10.3390/diagnostics12061302)
Supplement: Supplementary file 1 [file diagnostics-12-01302-s001.zip › diagnostics-1710999-supplementary files.pdf]

Supplementary appendix

This supplemental material has been provided by the authors to give readers additional information about their work.

Supplement to: Shim SR, Kim SJ, Hong MH, Lee JH, Kang MG, Han HW. Diagnostic performance of antigen rapid diagnostic tests, chest computed tomography, and lung point-of-care-ultrasonography for SARS-CoV-2 compared with RT-PCR testing: A systematic review and network meta-analysis.

Figure S1. Quality Assessment of Diagnostic Accuracy Studies 2 (QUADAS-2) Study Quality Summary

Figure S2. Network meta-analysis forest plot and SROC between nasopharyngeal swab and other swab for RDT

Table S1. Characteristics of the included studies

Table S2. Search queries

Table S3. SUCRA Values of 3 Different methods for diagnosis of SARS-CoV-2 infection by PCR

Table S1. Characteristics of the included studies

Study No.	First author year_subgroup	Country	Index test			Reference standard			Specimen site for RDT	Study population
				No. of positive	Sample size		No. of positive	Sample size		
1	Agullo 2021_NP	Spain	RDT	76	77	PCR	56	575	Nasopharyngeal	Age unclear
	Agullo 2021_nasal		RDT	59	59	PCR	73	600	Nasal	
	Agullo 2021_saliva		RDT	28	28	PCR	93	582	Saliva	
2	Ai 2020	China	CT	580	888	PCR	21	126	-	Adult
3	Albert 2021	Spain	RDT	43	43	PCR	11	369	Nasopharyngeal	Adult and children
4	Alemany 2020_NP	Spain	RDT	779	779	PCR	55	140	Nasopharyngeal	Adult and children
	Alemany 2020_nasal		RDT	93	98	PCR	24	389	Nasal	Adult and children
5	Asai 2021	Japan	RDT	49	53	PCR	14	252	Saliva	Age unclear
6	Aslan 2020	Turkey	CT	226	246	PCR	24	60	-	Adult
7	Barbosa 2020	Brazil	CT	23	48	PCR	2	43	-	Adult
8	Baro 2021_Panbio	Spain	RDT	39	40	PCR	62	246	Nasopharyngeal	Age unclear
	Baro 2021_Clinitest		RDT	52	55	PCR	49	231		
	Baro 2021_Standard Q		RDT	44	51	PCR	57	235		
	Baro 2021_Lepu		RDT	46	66	PCR	55	220		
	Baro 2021_Surescreen V		RDT	29	33	PCR	72	253		
9	Basso 2021	Italy	RDT	71	80	PCR	16	154	Nasopharyngeal	Adult
10	Beck 2021	USA	RDT	47	48	PCR	14	298	Nasopharyngeal	Adult and children
11	Bellini 2020	Italy	CT	80	156	PCR	62	416	-	Adult and children
12	Berger 2021_Panbio	Switzerland	RDT	106	106	PCR	18	429	Nasopharyngeal	Adult
	Berger 2021_Standard Q		RDT	170	171	PCR	21	358		Adult
13	Besutti 2020	Italy	CT	438	454	PCR	158	242	-	Adult
14	Bianchi 2021	Italy	US	120	184	PCR	20	176	-	Age unclear
15	Bosso 2020	Italy	US	19	22	PCR	7	31	-	Age unclear
16	Mboumba 2021	France	RDT	90	90	PCR	10	60	Nasopharyngeal	Age unclear
17	Brenner 2021	USA	US	79	102	PCR	13	72	-	Age unclear
18	Cartocci Gaia 2020	Italy	CT	147	171	PCR	15	143	-	Adult
19	Caruana 2021_Standard Q	Switzerland	RDT	47	48	PCR	67	484	Nasopharyngeal	Adult
	Caruana 2021_Panbio		RDT	47	49	PCR	67	483		
	Caruana 2021_Exdia		RDT	55	57	PCR	59	475		
	Caruana 2021_BD Veritor		RDT	47	48	PCR	67	484		
20	Caruso 2020	Italy	CT	60	102	PCR	2	56	-	Adult
21	Cerutti 2020_ER	Italy	RDT	75	75	PCR	29	110	Nasopharyngeal	Adult
	Cerutti 2020_travelers		RDT	2	2	PCR	3	143		
22	Chaimayo 2020	Thailand	RDT	59	64	PCR	1	390	Nasopharyngeal	Adult and children
23	Ciotti 2021	Italy	RDT	12	12	PCR	27	38	Nasopharyngeal	Adult and children
24	Colombi 2020	Italy	US	311	360	PCR	29	126	-	Adult
25	Debray 2020	France	CT	120	124	PCR	40	117	-	Adult
26	Dofferhoff 2020	Netherlands	CT	136	172	PCR	18	140	-	Adult
27	Dominguez 2021	Spain	RDT	19	19	PCR	1	11	Unclear	Adult
28	Drain 2021_ANT	UK/USA	RDT	81	87	PCR	2	170	Nasal	Adult and children
	Drain 2021_NP		RDT	39	44	PCR	1	211	Nasopharyngeal	
29	Ducray 2020	France	CT	259	308	PCR	28	386	-	Adult
30	Falaschi 2020	Italy	CT	419	485	PCR	43	288	-	Adult
31	Favresse 2021_Biotical	Belgium	RDT	64	65	PCR	32	123	Nasopharyngeal	Adult and children
	Favresse 2021_Panbio		RDT	65	65	PCR	31	123		
	Favresse 2021_Healgen		RDT	74	77	PCR	22	111		
	Favresse 2021_Roche		RDT	67	67	PCR	29	121		
	Favresse 2021_VITROS		RDT	80	80	PCR	16	108		
32	Fenollar 2021	France	RDT	154	161	PCR	50	180	Nasopharyngeal	Age unclear
33	Ferguson 2021	UK	RDT	2	2	PCR	6	718	Nasal	Adult
34	Fonsi 2020	Italy	CT	41	43	PCR	3	20	3-arm study of CT, US by PCR	Adult
	Fonsi 2020		US	30	34	PCR	3	20		
35	Fujioka 2020	Japan	CT	57	67	PCR	19	87	-	Adult
36	Gezer 2020	Turkey	CT	92	97	PCR	6	125	-	Adult
37	Giannitto 2020	Italy	CT	14	24	PCR	6	44	-	Adult
38	Gietema 2020	Netherlands	CT	74	109	PCR	9	84	-	Adult

39	Gili 2021_selected	Italy	RDT	86	97	PCR	9	129	Nasopharyngeal	Age unclear
	Gili 2021_screening		RDT	90	220	PCR	0	1518		
40	Gremmels 2020_Utrecht	Netherlands	RDT	101	101	PCR	38	1266	Nasopharyngeal	Adult
	Gremmels 2020_Aruba		RDT	51	51	PCR	12	157		
41	Guillo 2020	France	CT	103	114	PCR	26	100	-	Adult
42	Gupta 2020	India	RDT	63	64	PCR	14	266	Nasopharyngeal	Adult
43	Haak 2020	Netherlands	US	23	51	PCR	1	42	3-arm study of CT, US by PCR	Adult
	Haak 2020		CT	2	15	PCR	1	42		
44	He 2020	China	CT	26	28	PCR	8	54	-	Adult and children
45	Hermans 2020	Netherlands	CT	120	142	PCR	13	177	-	Adult
46	Hernigou 2020	Belgium	CT	13	15	PCR	3	32	-	Adult
47	Herpe 2021	France	CT	1999	2524	PCR	250	2300	-	Adult and children
48	Hirotsu 2021	Japan	RDT	37	37	PCR	3	992	Nasopharyngeal	Age unclear
49	Homza 2021_Ecotest	Czech	RDT	81	88	PCR	26	230	Nasopharyngeal	Adult
	Homza 2021_Joysbio		RDT	52	54	PCR	38	171		
	Homza 2021_ND COVID		RDT	54	104	PCR	23	87		
	Homza 2021_Standard Q		RDT	26	27	PCR	16	112		
	Homza 2021_VIVADiag		RDT	38	45	PCR	53	223		
50	Houston 2021	UK	RDT	242	264	PCR	38	464	Nasopharyngeal	Adult
51	Igloi 2021	Netherlands	RDT	158	162	PCR	28	808	Nasopharyngeal	Adult
52	Jaaskelainen 2021_Sofia	Finland	RDT	119	119	PCR	29	69	Nasopharyngeal	Age unclear
	Jaaskelainen 2021_Standard Q		RDT	128	128	PCR	30	70		
	Jaaskelainen 2021_Panbio		RDT	126	126	PCR	26	64		
53	Jalil et al2020	USA	US	31	34	PCR	5	35	-	Age unclear
54	James 2021	USA	RDT	86	89	PCR	66	2250	Nasal	Adult
55	Kannian 2021	India	RDT	15	15	PCR	12	22	Saliva	Age unclear
56	Kilic 2021	USA	RDT	77	92	PCR	39	1292	Nasal	Adult and children
57	Kohmer 2021_R-Biopharm	Germany	RDT	29	30	PCR	45	70	Nasopharyngeal	Age unclear
	Kohmer 2021_Standard Q		RDT	32	32	PCR	42	68		
	Kohmer 2021_Nadal		RDT	18	18	PCR	56	82		
	Kohmer 2021_LumiraDx		RDT	37	37	PCR	37	63		
58	Korevaar 2020	Netherlands	CT	117	142	PCR	9	97	-	Adult
59	Krdzalic 2020	Netherlands	CT	25	32	PCR	3	24	-	Adult
60	Kruttgen 2021	Germany	RDT	53	56	PCR	22	94	Nasopharyngeal	Age unclear
61	Kuzan 2020	Turkey	CT	48	69	PCR	21	51	-	Adult
62	Lambert-Niclot 2020	France	RDT	47	47	PCR	47	91	Nasopharyngeal	Age unclear
63	Lefever 2021	Belgium	RDT	138	138	PCR	66	276	Nasopharyngeal	Adult and children
64	Li 2020	China	CT	42	61	PCR	1	31	-	Adult
65	Lieveld 2020	Netherlands	US	79	108	PCR	7	78	-	Age unclear
66	Linares 2020	Spain	RDT	44	44	PCR	16	211	Nasopharyngeal	Adult
67	Lindner 2021_AN	Germany	RDT	29	31	PCR	10	258	Nasal	Adult
	Lindner 2021_NP		RDT	31	32	PCR	8	257	Nasopharyngeal	
68	Liotti 2021	Italy	RDT	49	53	PCR	55	306	Nasopharyngeal	
69	Luo 2020a	China	CT	26	40	PCR	4	33	-	Adult and children
70	Luo 2020b	China	CT	70	77	PCR	8	63	-	Adult
71	Mei 2020	USA	CT	274	313	PCR	145	592	-	Adult and children
72	Merino 2021	Spain	RDT	325	332	PCR	34	626	Nasopharyngeal	Adult and children
73	Mertens 2020	Belgium	RDT	76	77	PCR	56	251	Nasopharyngeal	Age unclear
74	Miranda 2020	Brazil	CT	30	31	PCR	6	44	-	Adult and children
75	Mockel 2021_adult	Germany	RDT	67	67	PCR	22	204	Nasopharyngeal	Adult
	Mockel 2021_children		RDT	18	19	PCR	7	183		Children
76	Nalumansi 2020	Uganda	RDT	63	76	PCR	27	186	Nasopharyngeal	Adult
77	Narinx 2020	Belgium	US	14	73	PCR	3	68	3-arm study of CT, US by PCR	Adult
	Narinx 2020		CT	12	22	PCR	3	68		
78	Okoye 2021	USA	RDT	24	24	PCR	21	2614	Nasal	Adult and children
79	Olearo 2020_Standard Q	Germany	RDT	41	41	PCR	43	143	Oropharyngeal	Age unclear
	Olearo 2020_Panbio		RDT	37	37	PCR	47	147		
	Olearo 2020_MEDsan		RDT	38	41	PCR	46	143		
	Olearo 2020_CLINITEST		RDT	46	46	PCR	38	138		

80	Osterman 2021_Standard F<br>Osterman 2021_Standard Q	Germany	RDT<br>RDT	115<br>165	123<br>174	PCR<br>PCR	74<br>91	426<br>468	Nasopharyngeal	Adult and children
81	Parada-Ricart 2021	Spain	RDT	19	40	PCR	7	132	Nasopharyngeal	Age unclear
82	Pare 2020	USA	US	24	31	PCR	3	12	-	Age unclear
83	Pekosz 2021	USA	RDT	27	30	PCR	1	221	Nasopharyngeal	Adult
84	Perez-Garcia 2021_CerTest<br>Perez-Garcia 2021_Panbio	Spain	RDT<br>RDT	91<br>102	91<br>102	PCR<br>PCR	79<br>68	229<br>218	Nasopharyngeal	Adult
85	Pilarowski 2020	USA	RDT	15	15	PCR	11	863	Nasal	Age unclear
86	Pivetta 2021	Italy	US	101	107	PCR	6	121	-	Age unclear
87	Pollock 2021	USA	RDT	226	238	PCR	66	2070	Nasal	Adult and children
88	Porte 2020	Chile	RDT	77	77	PCR	5	50	Nasopharyngeal	Adult and children
89	Prokop 2020	Netherlands	CT	43	48	PCR	10	57	-	Adult and children
90	Ristic 2021	Serbia	RDT	25	25	PCR	18	95	Nasopharyngeal	Adult and children
91	Rottenstreich 2021	Israel	RDT	5	5	PCR	4	1321	Nasopharyngeal	Adult
92	Salvagno 2021	Italy	RDT	108	109	PCR	41	212	Nasopharyngeal	Adult
93	Sberna 2021	Italy	RDT	5	5	PCR	57	131	Saliva	Adult
94	Schildgen 2021_Rapigen<br>Schildgen 2021_Panbio<br>Schildgen 2021_Standard Q	Germany	RDT<br>RDT<br>RDT	14<br>21<br>37	18<br>28<br>62	PCR<br>PCR<br>PCR	28<br>21<br>5	55<br>45<br>11	Bronchoalveolar lavage and throat wash	Age unclear
95	Schmid 2020<br>Schmid 2020	Germany	US<br>CT	30<br>7	52<br>15	PCR<br>PCR	9<br>9	83<br>83	3-arm study of CT, US by PCR	Age unclear
96	Schulze-hagen 2020	Germany	CT	65	81	PCR	4	110	-	Adult
97	Scohy 2020	Belgium	RDT	32	32	PCR	74	116	Nasopharyngeal	Adult and children
98	Seitz 2021	Austria	RDT	8	8	PCR	10	32	Saliva	Adult and children
99	Shrestha 2020	Nepal	RDT	40	40	PCR	7	73	Nasopharyngeal	Adult and children
100	Song 2020	China	CT	108	163	PCR	3	48	-	Adult
101	Sorlini 2020	Italy	US	264	298	PCR	23	86	-	Age unclear
102	Steuwe 2020	Germany	CT	19	38	PCR	0	67	-	Adult
103	Stokes 2021	Canada	RDT	231	233	PCR	37	1408	Nasopharyngeal	Adult and children
104	Takeuchi 2021	Japan	RDT	91	91	PCR	14	1095	Nasopharyngeal	Adult
105	Thakur 2021	India	RDT	29	30	PCR	55	647	Nasopharyngeal	Adult
106	Toptan 2020_Berlin<br>Toptan 2020_Frankfurt	Germany	RDT<br>RDT	45<br>16	45<br>16	PCR<br>PCR	13<br>16	22<br>54	Nasopharyngeal	Age unclear
107	Torres 2021_symptomatic<br>Torres 2021_asymptomatic	Spain	RDT<br>RDT	73<br>15	73<br>15	PCR<br>PCR	18<br>10	105<br>77	Nasopharyngeal	Adult and children
108	Torres 2021	Spain	RDT	38	38	PCR	41	596	Nasopharyngeal	Adult and children
109	Tung 2021	Spain	US	39	51	PCR	15	38	-	Adult
110	Turcato 2021	Italy	RDT	179	209	PCR	44	3201	Nasopharyngeal	Age unclear
111	Veyrenche 2021	France	RDT	13	13	PCR	32	52	Nasopharyngeal	Adult
112	Villaverde 2021	Spain	RDT	35	38	PCR	42	1582	Nasopharyngeal	Children
113	Volpicel 2021	EU multinational	US	921	1130	PCR	101	332	-	Adult and children
114	Wang 2020	China	CT	28	61	PCR	1	129	-	Adult and children
115	Weitzel 2020_Rapigen<br>Weitzel 2020_LimingBio<br>Weitzel 2020_Savant<br>Weitzel 2020_Bioeasy	Chile	RDT<br>RDT<br>RDT<br>RDT	49<br>0<br>13<br>68	49<br>1<br>13<br>68	PCR<br>PCR<br>PCR<br>PCR	30<br>9<br>65<br>12	60<br>18<br>96<br>43	Nasopharyngeal	Adult
116	Xiong 2020	China	CT	19	27	PCR	1	20	-	Adult and children
117	Young, B.C 2021	UK	RDT	133	133	PCR	81	653	Nasopharyngeal	Adult
118	Young, S 2020	USA	RDT	29	30	PCR	9	221	Nasopharyngeal	Adult

Figure S1. Quality Assessment of Diagnostic Accuracy Studies 2 (QUADAS-2) Study Quality Summary

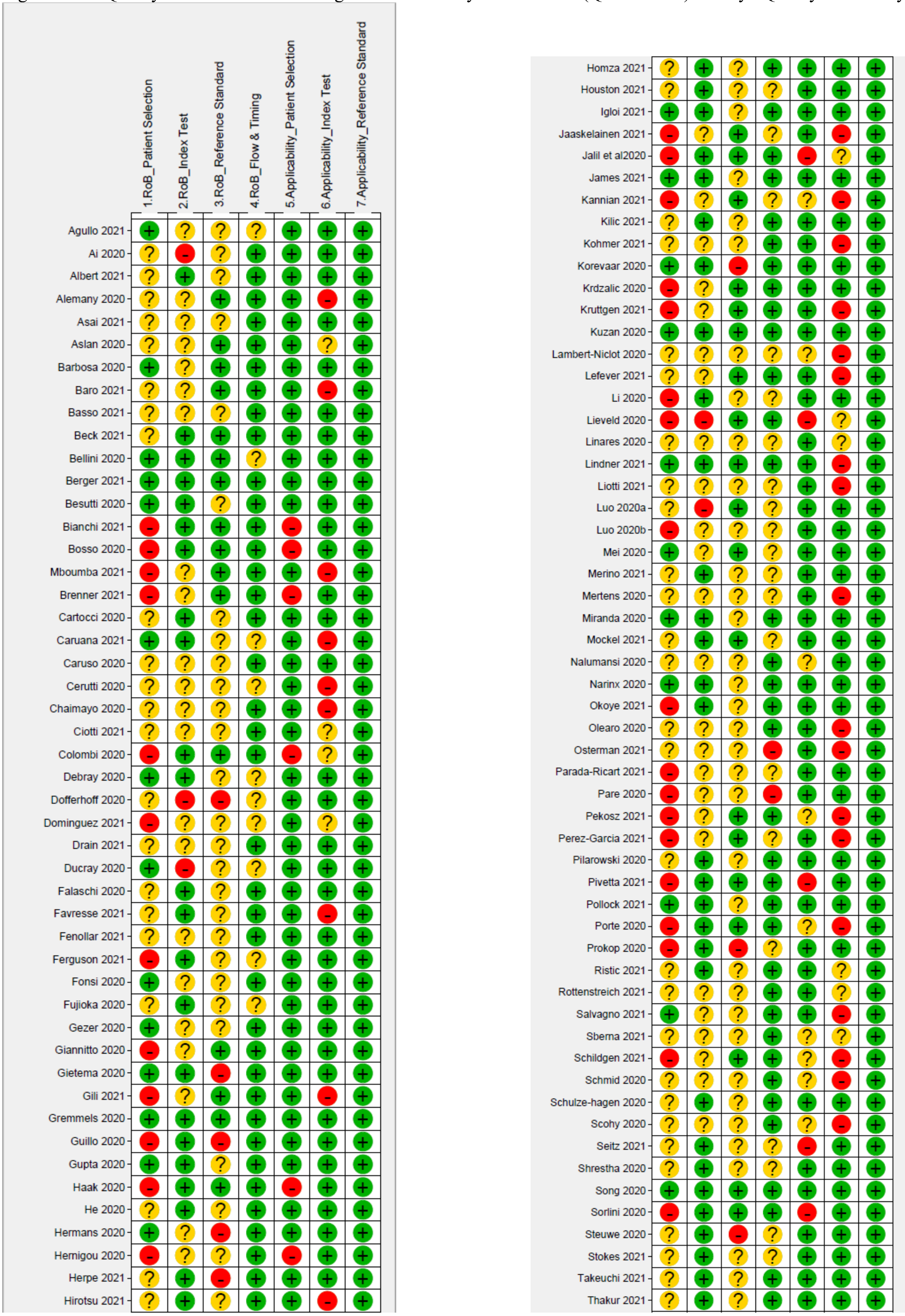

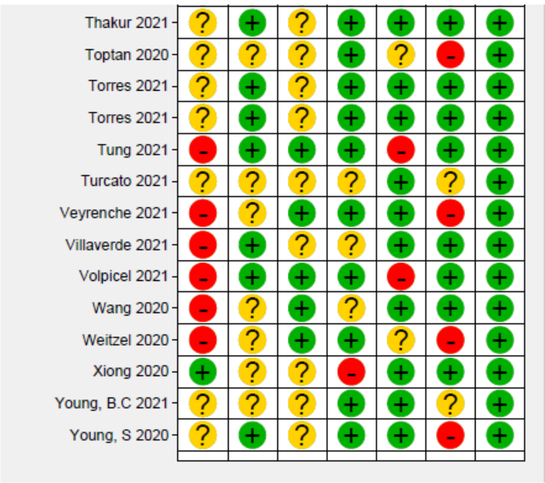

Thakur 2021
Toptan 2020
Torres 2021
Torres 2021
Tung 2021
Turcato 2021
Veyrenche 2021
Villaverde 2021
Volpicel 2021
Wang 2020
Weitzel 2020
Xiong 2020
Young, B.C 2021
Young, S 2020

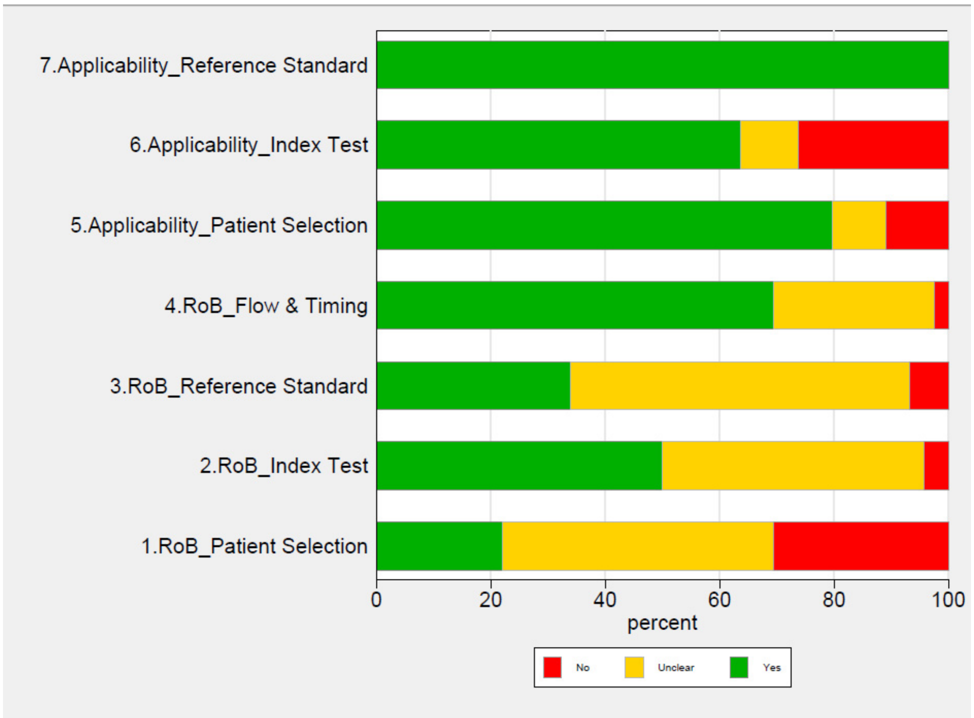

7.Applicability_Reference Standard
6.Applicability_Index Test
5.Applicability_Patient Selection
4.RoB_Flow & Timing
3.RoB_Reference Standard
2.RoB_Index Test
1.RoB_Patient Selection
0
20
40
60
80
100
percent
No
Unclear
Yes

Figure S2. Network meta-analysis forest plot and SROC between nasopharyngeal swab and other swab for RDT

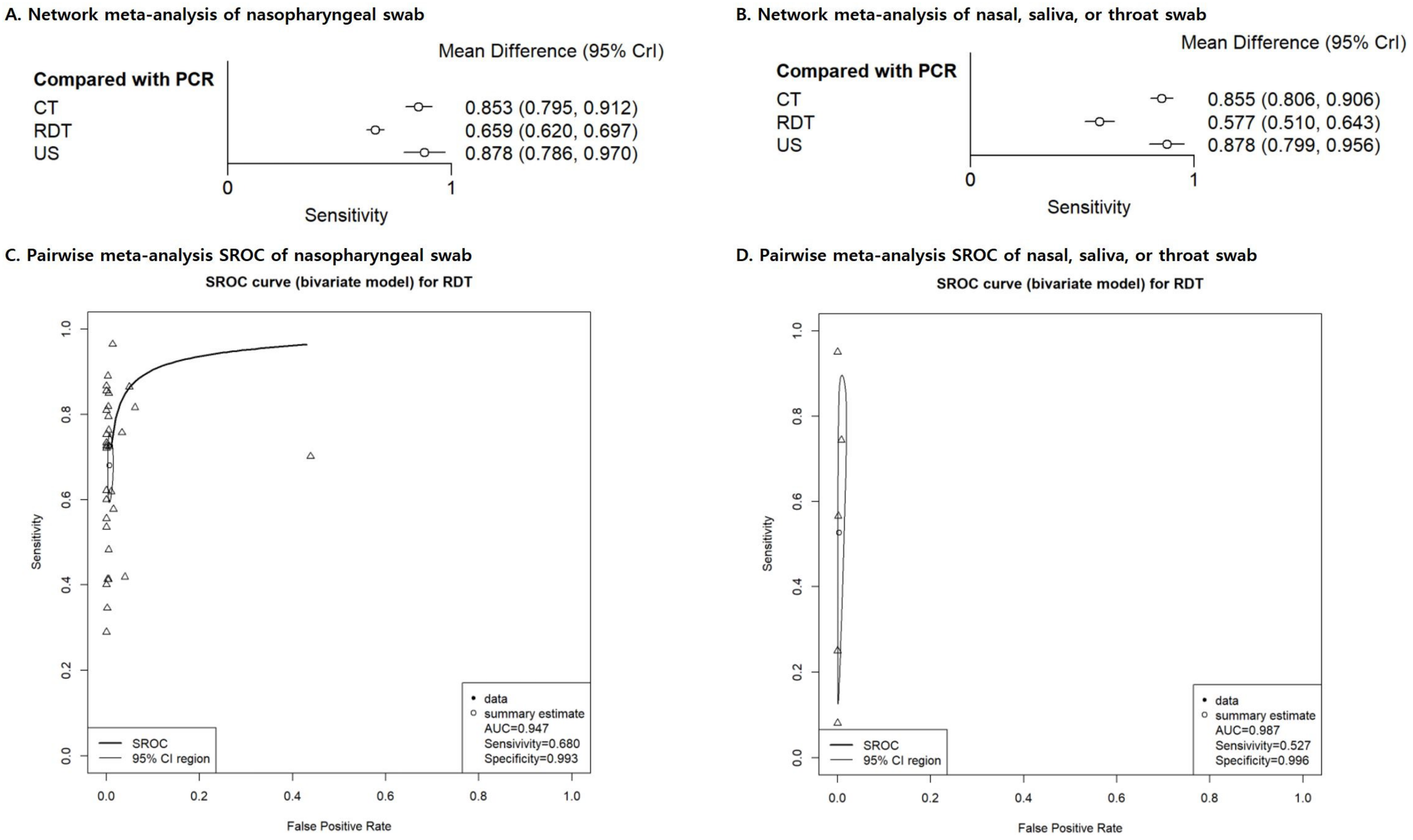

Table S2. Search queries

PubMed and Cochrane Library

1. Disease
("SARS-CoV-2"[Mesh] OR "COVID-19"[Mesh] OR "SARS-CoV-2 variants" [Supplementary Concept] OR "COVID-19 breakthrough infections" [Supplementary Concept] OR "COVID-19"[tiab] OR "COVID 19"[tiab] OR "COVID19"[tiab])

2. Index tests and reference standard
("COVID-19 testing"[MeSH terms] OR "COVID 19 Test*"[tiab]) AND ("rapid test*"[tiab] OR "quick test*"[tiab] OR "antigen test*"[tiab] OR "STANDARD Q"[tiab] OR "PANBIO"[tiab] OR "point-of-care"[tiab] OR "Reverse Transcriptase Polymerase Chain Reaction"[Mesh] OR "RT-PCR"[tiab] OR "Tomography, X-Ray Computed"[Mesh] OR "Computed tomography"[tiab] OR "Ultrasonography"[Mesh] OR "Ultrasonography"[tiab])

Embase

1. Disease
('severe acute respiratory syndrome coronavirus 2'/exp OR 'coronavirus disease 2019'/exp OR 'covid-19':ti,ab OR 'covid 19':ti,ab OR 'covid19':ti,ab)

2. Index tests and reference standard
('covid-19 testing'/exp OR 'covid 19 test*':ti,ab) AND ('rapid test*':ti,ab OR 'quick test*':ti,ab OR 'antigen test*':ti,ab OR 'standard q':ti,ab OR 'panbio':ti,ab OR 'point-of-care':ti,ab OR 'reverse transcription polymerase chain reaction'/exp OR 'rt-pcr':ti,ab OR 'x-ray computed tomography'/exp OR 'computed tomography':ti,ab OR 'echography'/exp OR 'ultrasonography':ti,ab)

Table S3. SUCRA Values of 3 Different methods for diagnosis of SARS-CoV-2 infection by PCR

Diagnosis	SUCRA values				
	Sensitivity	Specificity	PPV	NPV	Accuracy
US	0.893	0.334	0.461	0.674	0.385
CT	0.774	0.666	0.539	0.891	0.657
RDT	0.333	1.000	1.000	0.434	0.957

Abbreviations: RDT, rapid antigen detection tests; CT, computed tomography; US, ultrasonography, PPV, positive predictive value; NPV, negative predictive value.
